# Supplementary material for: Deletion of wfs1 Impairs Oligodendrocyte Precursor Cells Dorsal Distribution and Myelination Through the wfs1-hmgcs1 Axis in Zebrafish
Source: Biology (Basel). 2026 Aug 16;15(16):1402. doi: 10.3390/biology15161402 (PMC13510151; doi:10.3390/biology15161402)
Supplement: Supplementary file 1 [file biology-15-01402-s001.zip › File S1-Transcriptome sequencing data and differentially expressed genes (DEGs) in wfs1-- zebrafish at 5 dpf/2.KEGG/wfs1KOvsWT/GSEA/wfs1KO/FOLATE_BIOSYNTHESIS/FOLATE_BIOSYNTHESIS(DRE00790).html]

Details for gene set FOLATE\_BIOSYNTHESIS(DRE00790)[GSEA]

|  || Dataset | deg.sample |
| Phenotype | sample.cls |
| Upregulated in class | wfs1KO |
| GeneSet | FOLATE\_BIOSYNTHESIS(DRE00790) |
| Enrichment Score (ES) | 0.33777258 |
| Normalized Enrichment Score (NES) | 1.326914 |
| Nominal p-value | 0.15650406 |
| FDR q-value | 0.26357913 |
| FWER p-Value | 0.946 |
Table: GSEA Results Summary

  

Fig 1: Enrichment plot: FOLATE\_BIOSYNTHESIS(DRE00790)      
 Profile of the Running ES Score & Positions of GeneSet Members on the Rank Ordered List

  

| PROBE | DESCRIPTION (from dataset) | GENE SYMBOL | GENE\_TITLE | RANK IN GENE LIST | RANK METRIC SCORE | RUNNING ES | CORE ENRICHMENT || 1 | 791448\_1 | qdprb1 |  |  | 911 | 2.212 | 0.0613 | Yes |
| 2 | 557552 | zgc:153031 |  |  | 1026 | 2.071 | 0.1419 | Yes |
| 3 | 100330410 | alp3 |  |  | 1139 | 1.962 | 0.2182 | Yes |
| 4 | 81882 | dhfr |  |  | 1508 | 1.649 | 0.2737 | Yes |
| 5 | 574009 | alpi.2 |  |  | 1560 | 1.616 | 0.3378 | Yes |
| 6 | 352943\_1 | tph1a |  |  | 3540 | 0.921 | 0.3131 | No |
| 7 | 791448 | qdprb1 |  |  | 5012 | 0.680 | 0.2946 | No |
| 8 | 337696 | cbr1l |  |  | 5935 | 0.594 | 0.2898 | No |
| 9 | 30384 | th |  |  | 6648 | 0.524 | 0.2887 | No |
| 10 | 100073330 | pts |  |  | 6682 | 0.521 | 0.3089 | No |
| 11 | 414844 | th2 |  |  | 7591 | 0.448 | 0.2986 | No |
| 12 | 415103 | tph1b |  |  | 7790 | 0.438 | 0.3102 | No |
| 13 | 795445 | sprb |  |  | 8292 | 0.413 | 0.3112 | No |
| 14 | 373866 | cbr1 |  |  | 9333 | 0.345 | 0.2926 | No |
| 15 | 406624 | ggh |  |  | 11063 | 0.241 | 0.2481 | No |
| 16 | 393787 | pcbd1 |  |  | 13059 | 0.137 | 0.1911 | No |
| 17 | 541539 | alpi.1 |  |  | 13250 | 0.128 | 0.1903 | No |
| 18 | 100192219 | gch1 |  |  | 13304 | 0.125 | 0.1937 | No |
| 19 | 553452 | si:dkey-180p18.9 |  |  | 14479 | 0.070 | 0.1597 | No |
| 20 | 378962 | pah |  |  | 15203 | 0.037 | 0.1386 | No |
| 21 | 555470 | gphna |  |  | 16217 | -0.008 | 0.1071 | No |
| 22 | 100331902 | mocos |  |  | 16432 | -0.016 | 0.1010 | No |
| 23 | 407712\_1 | tph2 |  |  | 16433 | -0.016 | 0.1016 | No |
| 24 | 563836 | zgc:171566 |  |  | 17791 | -0.067 | 0.0618 | No |
| 25 | 64263 | gch2 |  |  | 19886 | -0.161 | 0.0026 | No |
| 26 | 793471 | mocs1 |  |  | 20932 | -0.212 | -0.0216 | No |
| 27 | 30384\_1 | th |  |  | 23375 | -0.353 | -0.0840 | No |
| 28 | 553228 | LOC553228 |  |  | 25774 | -0.509 | -0.1386 | No |
| 29 | 566323 | qdpra |  |  | 25822 | -0.512 | -0.1192 | No |
| 30 | 554136 | spra |  |  | 26002 | -0.529 | -0.1033 | No |
| 31 | 352943 | tph1a |  |  | 26886 | -0.618 | -0.1059 | No |
| 32 | 791448\_2 | qdprb1 |  |  | 27072 | -0.637 | -0.0858 | No |
| 33 | 407712 | tph2 |  |  | 27844 | -0.721 | -0.0807 | No |
| 34 | 406746\_1 | fpgs |  |  | 28002 | -0.742 | -0.0555 | No |
| 35 | novel.687 | - |  |  | 28540 | -0.829 | -0.0386 | No |
| 36 | 393982 | alpl |  |  | 29631 | -1.104 | -0.0279 | No |
| 37 | 100007749 | gphnb |  |  | 29678 | -1.121 | 0.0162 | No |
| 38 | 415103\_1 | tph1b |  |  | 30140 | -1.309 | 0.0549 | No |
Table: GSEA details [plain text format]

  

Fig 2: FOLATE\_BIOSYNTHESIS(DRE00790)      
 Blue-Pink O' Gram in the Space of the Analyzed GeneSet

  

Fig 3: FOLATE\_BIOSYNTHESIS(DRE00790): Random ES distribution      
 Gene set null distribution of ES for **FOLATE\_BIOSYNTHESIS(DRE00790)**
